# Selected Determinants of Machines and Devices Standardization in Designing Automated Production Processes in Industry 4.0

**DOI:** 10.3390/ma16010312

**Published:** 2022-12-29

**Authors:** Piotr Kuryło, Adam Wysoczański, Joanna Cyganiuk, Maria Dzikuć, Szymon Szufa, Piotr Bonarski, Anna Burduk, Peter Frankovský, Piotr Motyka, Daniel Medyński

**Affiliations:** 1 Faculty of Mechanical Engineering, University of Zielona Góra, Licealna Street 9, 65-417 Zielona Góra, Poland; 2 Faculty of Economics and Management, University of Zielona Góra, Licealna Street 9, 65-417 Zielona Góra, Poland; 3 Faculty of Process and Environmental Engineering, Lodz University of Technology, Wolczanska Street 213, 90-924 Lodz, Poland; 4 Faculty of Managemant, Wrocław University of Science and Technology, Łukasiewicza Street 5, 50-370 Wrocław, Poland; 5 Faculty of Mechanical Engineering, Wrocław University of Science and Technology, Łukasiewicza Street 5, 50-370 Wrocław, Poland; 6 Department of Applied Mechanics and Mechanical Engineering, Faculty of Mechanical Engineering, Technical University of Košice, Letná 9, 040 02 Košice, Slovakia; 7 Faculty of Technical and Economic Science, Witelon Collegium State University, Sejmowa Street 5A, 59-220 Legnica, Poland

**Keywords:** synergy and coherence, production process, standardization, industry 4.0, internet of things

## Abstract

The study presents a practical application of multi-criteria standardization of machines and devices in the design of the automated production processes in industry 4.0 and its direct impact on the economic aspects of an enterprise, along with a comparison of the state before and after the implementation of the proposed changes. The solutions recommended in the article also fit into the assumptions of low-carbon development by implementing solutions that reduce energy consumption. The research carried out and presented in the text confirmed the effectiveness of the described solution. The study also presents examples confirming the correctness of implementing standardization, synergy and coherence in the design of production processes. Additionally, a new advanced eLean application was presented to support production processes in the field of Lean Management. The Total Productive Maintenance (TPM) module currently implemented in the industry is concerned with ensuring the maximum efficiency of machines and devices.

## 1. Introduction

The contemporary design and implementation of a production process should take into account all the elements of a given technology used in this type of process and affecting the quality of the finished product, the speed of implementation of a specific production process, the time of order fulfillment and, consequently, the price of the finished product. The design and implementation of the production process itself should be carried out according to specific criteria.

Technical preparation of production must be preceded by a full analysis of the construction documentation from the point of view of standardization, unification, rational selection, material savings, optimal shaping of semi-finished products and processing technology of individual parts [1]. Such analysis should take into account the impact of the technology used in the production process on the surrounding environment within which the production process is to be carried out [2,3,4].

The optimization of the design process itself should be based on the criteria adopted in advance, according to which the production of the previously designed products will be implemented. It is, therefore, necessary to introduce optimization and systematization already in the conceptual phase of the project. It plays a significant role in reducing the number of media involved in the production process. The subject analysis should therefore consider three fundamental criteria, i.e., cost, time and energy criteria [5,6,7].

The analysis should consider the dependencies between the number of the adopted criteria and the optimal number of the adopted variants of implementing a given production technology with defined synergy and coherence factors between the parameters according to which the technology is implemented.

One of the basic elements (variants) of multi-criteria design of technological and production processes in the fourth industrial revolution is the standardization of the elements used, for example, when designing a technological line. Each designer of a production line, when selecting the consumable parts used, should, first of all, perform an analysis of the sales market of the machines and devices they intend to use for the production process. The conducted analysis allows for defining the availability and the possibility of purchasing spare parts as well as defining the required number of specialists who will have the knowledge and experience in the implementation of similar projects [8,9].

Apart from the necessity of standardization and the possibility of using frequently interchangeable elements of production lines, an important, if not the most important, aspect of the implementation of each production and technological process is the economic aspect, and the greatest loss for each production enterprise is the lost profits. The main problem causing the loss is the production line downtime. That is why the level of availability of spare parts is so important. If spare parts of a given type (manufacturer) are available on the local market, it allows for minimizing the delivery time and starting the production line in an emergency to the necessary minimum, which in turn shortens the downtime of the machine and does not generate unnecessary costs [10,11,12,13].

Obviously, the ideal solution for eliminating losses related to machine downtime is to create and maintain a spare parts warehouse, define a list of necessary elements based on technical and operational documentation (DTR), introduce procedures for determining stock levels (minimum, optimal) and ongoing supervision over the introduced standards. However, the experience shows that this is not possible in every situation. There are also non-standard situations (damage to an undefined element) that force technical departments to run an emergency procedure. Then, the race against time and the availability of individual elements begins, in which the availability of the local market plays the main role.

Another equally important problem, such as the availability of spare parts, which affects the time to start up an inefficient production line, is the availability of specialists with knowledge of various types of damaged elements [14,15].

An example of the impact of the availability of spare parts at the time of launching a production line may be the standard of Programmable Logic Controllers (PLC) used in the technological processes of a specific manufacturer X. Most specialists in a given country may have comprehensive knowledge and experience in diagnostics and programming of controllers of a given manufacturer X. An incorrect approach in the design process is a proposal to use the manufacturer Y controller. This may cause unnecessary and time-consuming complications and the prolongation of activities related to the launch of production due to, for example, the differences in input modules or incompatibility of automation components [16,17,18].

Therefore, when designing each machine for the selected market, one should always perform a careful market analysis and define the standards that the manufacturer of automation components will follow.

Another very important element that should be taken into account when designing a production line is cooperation and setting standards related to other producers of a technological line (if the manufacturer is not the only contractor). When designing, for example, a two-section production line, the manufacturer of the first section may use the industrial automation components of manufacturer X, while the manufacturer of the second section may use the automation components of manufacturer Y. The entire production line should be integrated and combined into one integral whole. An important aspect here is the full compatibility of the designed sections, which involves additional work, and financial outlays to fully integrate and connect the two sections [19,20,21]. After analyzing the available literature, the need to address the problem of standardization in the design of production machines and devices for plants implementing Industry 4.0 production was defined. The goal is to standardize selected elements defined in the manuscript and then to test how standardization will affect the technological and economic aspects of the company.

## 2. Industry 4.0 and Multi-Criteria Design of Production Processes

The fundamental question of modern industry is: How do the assumptions of industry 4.0 translate into multi-criteria designing of technological and production processes?

The basic assumption of the industry 4.0 operation is to strengthen the synergy between individual elements of technological processes. Production lines should cooperate with robots (or other automated elements of the production system) dealing, for example, with loading the input material or receiving the final product, together with simultaneous communication with autonomous trolleys delivering material for production and receiving finished semi-finished products or products. This means that the main assumption of the fourth industrial revolution is to increase the possibility of using data in order to improve production processes through prediction and adaptation, limit human activities in places dangerous or harmful to health to the minimum possible, fill vacancies (in hazardous places) with machines and to strengthen communication between machines and devices in such a way that they become as autonomous as possible (Internet of things) using appropriate computer networks; smart, eclectic networks; or the Internet. With the help of the Internet (or a computer network), it is possible to exchange information directly or indirectly between devices. Such solutions are used, for example, in a household where household appliances “communicate with each other” to perform certain activities. Communication between devices as elements of the production system has already been used by richer and more developed production companies. The purpose of such communication is to synchronize the work of many elements of technological and production processes, which, for example, use industrial robots on the production line, autonomous trolleys for transport between stations and automated high storage racks [18,22,23,24,25]. The task of such synchronization is to eliminate the human factor, and it mainly concerns possible mistakes that a human may make in both production and technological processes. The task of synchronization is also to increase the efficiency and quality of processes.

**Figure 1:**
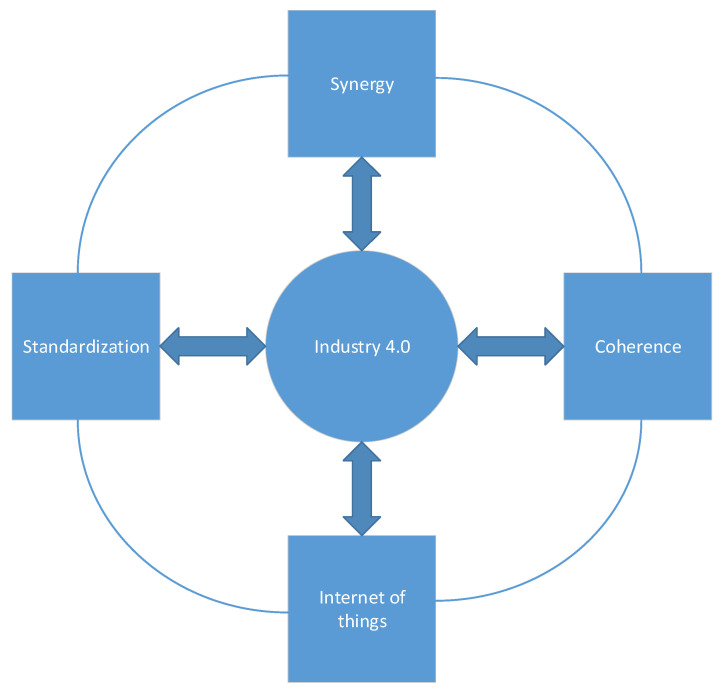
Structure of dependencies for industry 4.0.

The connection of the Internet of things with industry 4.0 is shown in Figure 1, where, in addition to the use of the Internet, there is also an emphasis on the synergy and coherence of production and technological processes.

Industry 4.0 should rely heavily on the synergy and multi-criteria nature of machines and technological processes in order to fully implement its initial assumptions. The unquestionable advantage of introducing the 4.0 system is the development of technology and reducing the losses of production companies, while the following question arises: what are the disadvantages and problems when implementing industry 4.0? Then, it would be necessary to analyze the costs of implementing automation and robotization of production lines as well as to consider what current work machines could replace human activities (operator, programmer, constructor). Table 1 presents a SWOT (Strengths, Weaknesses, Opportunities, Threats) analysis of the introduction of the fourth industrial revolution in manufacturing companies [26,27,28,29,30,31].

## 3. Materials and Methods

**Figure 2:**
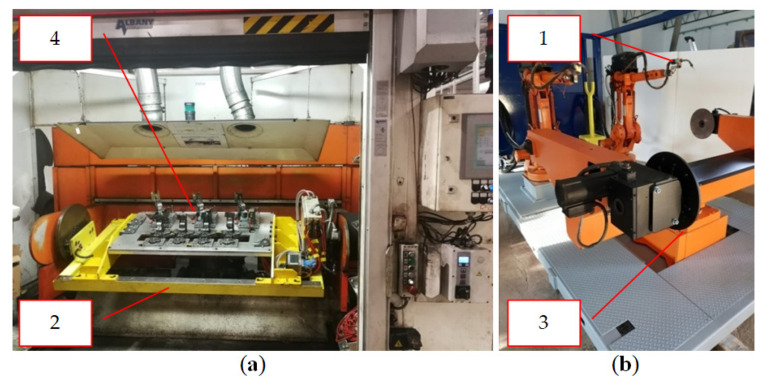
Welding cell: (**a**) view from the operator’s side; (**b**) view from the robots’ side (executive part of the cell).

The macroscopic observations showed that the ceramic particles were fully infiltrated with liquid metal during casting in the reinforcement zones. This is documented with the photos of the fragments of surfaces perpendicular and parallel to the reinforcement axis of the casting presented in Figure 2. In both cases, the complete continuity of the structure is visible at the interface between the matrix and the ceramic particles.

The main purpose of the study was to analyze and determine the impact of standardization, synergy and coherence in the design of machines and production devices on the economic aspects of a production company. The subject of the research was an automated/robotic production line. These studies were intended to answer the following questions:

What is the impact of the standardization of components (parts) used in the design of the technological line on the economic aspects of an enterprise?

What is the impact of the use of coherence and synergy in the design of production lines on increasing the efficiency of a production company?

What is the impact of the use of standardization during the technology design stage on the subsequent reduction in failure rates and the reduction in the average failure removal time rate at the time of using machines and production equipment?

The use of synergy, coherence and standardization in the process of designing production lines will reduce the costs associated with maintaining the full efficiency of production machines and equipment and training technical personnel, which will result in the improvement of the company’s financial indicators.

In the conducted research, three research methods were used, i.e., the observational method, the method of examining documents and the method of individual case analysis. By using the observational method as one of the oldest research methods, the entire machine park was reviewed in terms of energy and cost consumption. The organizational structure of the studied enterprise was reviewed, and the research problem was formulated. In the method of document examination, the analysis of financial indicators was performed before introducing the recommended changes, and the results were entered into the table (Table 2); then, one selected element (inverter) was standardized in the spare parts warehouse, and another analysis was performed. After the introduced standardization, the results were recorded in the table (Table 2), in which the comparison and summary of the cost reduction after the changes were made. This analysis confirmed the initial assumptions. It allowed us to proceed to the next research steps, i.e., standardization of a larger assortment group: 10%, 30% and 100% of the assortment possible to standardize. These results are presented in Table 3, and a summary was made. It confirmed the thesis. In the method of individual cases, an analysis of the problem of synergy and coherence in the technology used in the tested plant, and in particular, the issues of the automated welding process, was carried out.

The use of synergy, coherence and standardization phenomena during the process of designing production lines will reduce the costs associated with maintaining the full efficiency of machines and production equipment and technical training personnel, which in turn will improve the company’s financial indicators.

## 4. The Problem of Synergism in the Technology of Automated Welding Processes

The technological synergy of a process is a basic element of any automation process, and even more, the robotization of production lines. The first step in designing an automated technological line is to enforce mutual synergy between individual elements of a technological process.

**Figure 3:**
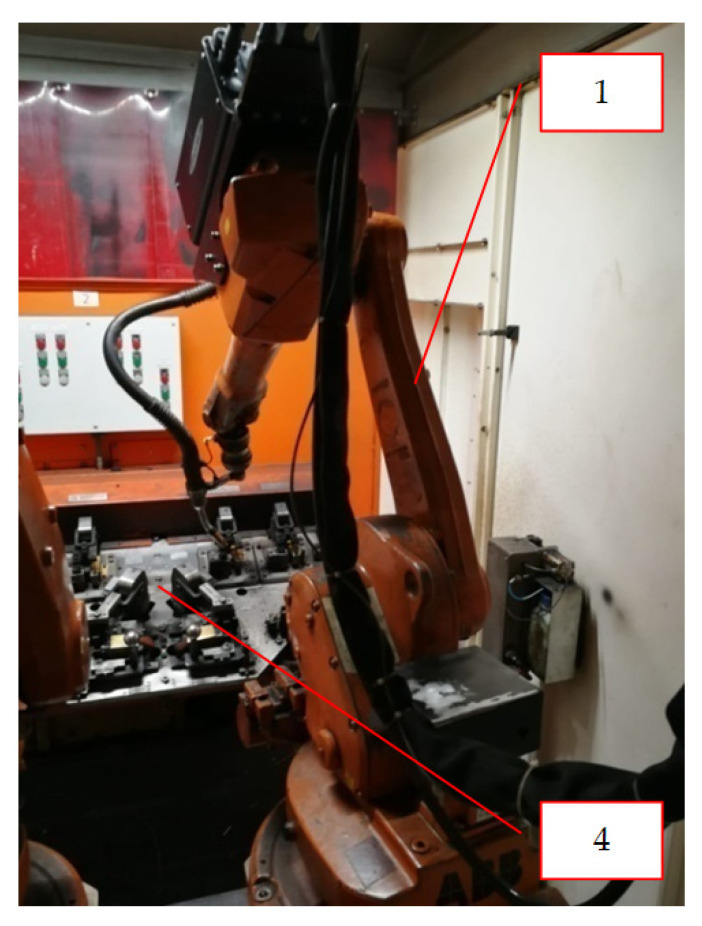
Welding cell—view from the robot’s side during the welding cycle.

A very good example to discuss this issue is the synergy of elements of a robotic welding cell. When designing and building such a cell, the main element is creating the synergy between the manipulator (item 1, Figure 2 and Figure 3) and the tool and the welding table (item 2, Figure 2). While an industrial robot performs welding operations, the welding table and the turntable (item 3, Figure 2 and Figure 3) change their positions in order to facilitate the robot’s access to the sensitive places of the welded element in the shortest time (item 4, Figure 2 and Figure 3).

It is the complete synergy that enables the welding operation to be performed. Another element is a turntable on which the welding tables are mounted. After the welding process is completed on the first table (Figure 3), a signal is sent from the robot that allows the table to return to the “0” position (initial position). If the table obtains the required position, another signal is generated that allows the release of the “bolt” and the commencement of the turntable operation, which makes a rotary movement causing the tables to be changed on the robot side. The welding table with the finished (welded) element returns to the operator, and then the other table enters the welding cell for the subsequent operations. Without the use of the technological synergy between the described elements (robot-welding table-rotator), it would not be possible to make a robotic welding cell [32,33,34,35,36,37,38].

**Figure 4:**
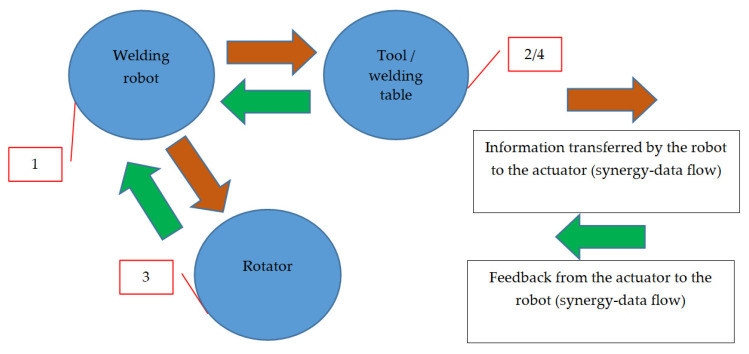
Synergy during the welding process.

The diagram of the operation of the discussed system, shown in Figure 2 and Figure 3, together with a demonstration of the elements that work in synergy during the welding process, is shown in Figure 4. The presented drawing (Figure 4) shows the way of information flow between the robot (item 1) and the execution elements, i.e., the rotator (item 3) and the welding table (item 2) on which the turntable (item 4) is mounted.

Automation and robotization are inseparable elements of the implementation of the ideology of industry 4.0; therefore, the level of technological synergy plays a key role here. The main element when designing technological processes based on the assumptions of the fourth industrial revolution is forcing synergy between all elements in the automated production area.

**Figure 5:**
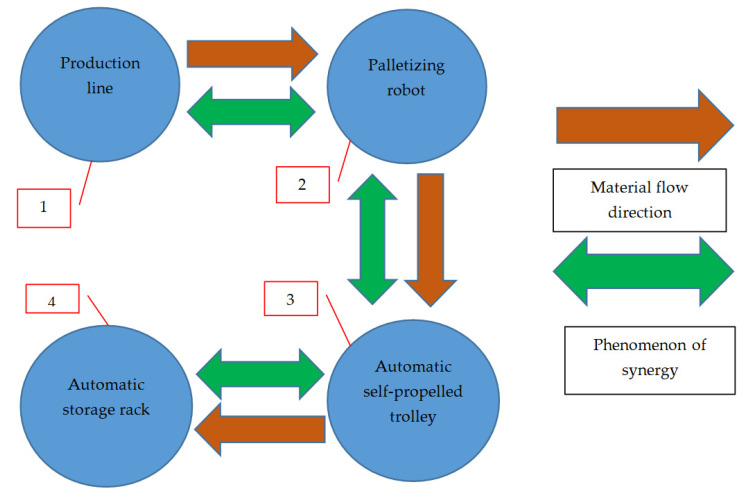
Material flow and synergy between machines.

An example of technological synergy in the design of technological processes based on the fourth industrial revolution is the design of an automated production line (in the analyzed case—a welding cell—Figure 2 and Figure 3) producing a steel structure. The presented production line (item 1, Figure 5), with a high degree of automation, should additionally have full synergy with the palletizing robot (item 2, Figure 5), which should be installed at the end of a technological line in order to pick up the manufactured elements and place them on pallets according to the given algorithms. After the robot completes the palletization, an automatic self-propelled trolley (item 3), which transports the manufactured elements to the warehouse (item 4, Figure 5), will be called. After delivering the items to the designated storage area (selected rack), the self-propelled trolley sends a signal to the storage rack that it is at the designated location. Subsequently, when the empty rack receives a given signal, it goes to the designated place and sends the information to the manipulator located on the platform in the warehouse zone that it is ready to load the semi-finished products brought by the trolley [39,40,41,42,43]. When the robot receives a signal from the rack, it will move on the platform to the designated place and start the process of reloading from the self-propelled trolley to the rack. After the reloading process is completed, the manipulator on the platform will move away from the rack and give a signal that the operation is completed. Then, the rack will return to its initial position with the “loaded” status, and the self-propelled trolley will start another cycle of collecting the finished products from the welding cell while the unloading manipulator will wait for the next tasks (Figure 5).

The production system designed in this way is a very good example of the synergy between technological elements in industry 4.0 (production line, production line manipulator, automatic self-propelled trolley, automatic rack and manipulator on the platform in the warehouse area) [44]. The scheme of operation of such a system with the marked elements that are in synergy is shown in Figure 5. It presents the direction of material flow in the production process, starting from the production line (item 1) to the palletizing robot (item 2), and then the automatic self-propelled trolley (item 3) delivers the product to the automatic storage rack (item 4).

Technological synergy is, therefore, a key element in the design and construction of plants based on the assumptions of the fourth industrial revolution. All items located in the automated and robotic production lines must be based on the main assumptions of technological synergy.

## 5. Synergism in the Structure of the Technological Process—Case Study

The conducted research included the analysis of technical design based on a robotic welding cell. By analyzing the literature [45], a new comprehensive structure of the technical design of a technological process was proposed. The recommended structure introduces, among other things, an analysis of the mutual influence of the technology used already at the design stage as an indispensable component [46,47].

The designed structure of a welding cell became the “starting point” in the analysis of the synergy coefficients and the determination of the coherence coefficients. Table 2 presents selected examples before and after the standardization performed in one of the automotive industry manufacturing companies on a selected warehouse item, which was an inverter. The analysis was performed on the inverters marked as X and Y interchangeably used in the production line, differing in parameters (supply voltage, admissible fluctuations in supply voltage, power demand, installation method) and on the inverters with the same technical parameters (recommended motor power, inverter power, rated output current, output voltage, current overload) [40,48,49].

If a company is standardized and uses one of the manufacturers’ inverters on all its machines, then it will not be forced to “freeze” financial capital into unnecessary storage of inverters. Therefore, it is possible to reduce the amount of the stored elements, and as a result, the associated costs will be reduced to a minimum, i.e., by 50%.

When analyzing the data included in Table 2, the company, by introducing standardization, saved EUR 1600 on two types of inverters that are unnecessarily kept in stock.

In turn, Table 3 shows an example using standardization for 150 stock items, which is 30% of the stock in a manufacturing company from the automotive industry employing 300 people and having a diversified technical assortment. Standardization applies to products such as spare parts for machines and production devices for welding purposes, assembly machines and injection molding machines.

After the introduction of standardization for 30% of the product range, it was possible to save approx. 5263 EUR/month (Table 3), which allows the company to save approx. EUR 63 thousand per year. Assuming that the trend will be maintained, the company will standardize the entire warehouse; the estimated savings could be approx. 210.5 thousand EUR/year with 100% of standardization (Table 3).

As can be seen in Table 2 and Table 3, standardization has a positive effect on the economic aspects of the enterprise. It is also one of the basic elements of the multi-criteria technical design structure and is usually introduced when determining technological or operational requirements, i.e., at the stage of optimization of automated production/technological processes.

This is the initial stage of the multi-criteria technical design, during which a market analysis should be performed, for which a given technology (technological line) or the entire production process is being prepared (designed).

**Figure 6:**
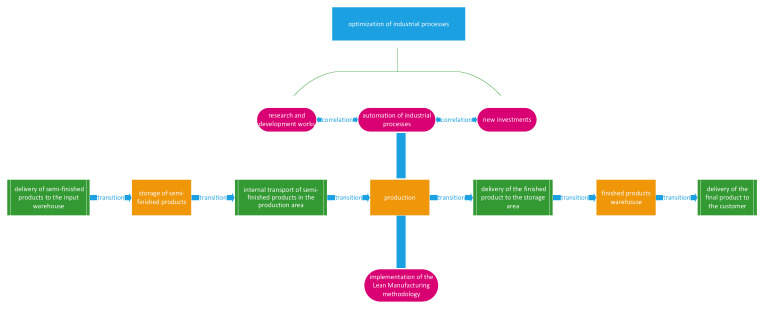
Application of optimization in production processes.

The optimization of production and technological processes leads to the creation of certain mathematical models, the components of which, i.e., sets of feasible solutions and optimization criteria, should reflect the leading concepts shaping the company’s policy. Optimization depends on the existence of a set of alternatives and can be associated with many elements of a production process (Figure 6). The implemented optimization must also take into account synergism, technology coherence and standardization at every stage of their implementation. Figure 6 shows an outline of a technological process and its successive elements, each other as well as the correlation of research and development with the automation of industrial processes and planning new investments, which, when combined with the simultaneous implementation of the Lean Manufacturing system, will result in optimization in the production area.

## 6. Discussion

The results presented in Table 2 and Table 3 confirm the hypothesis that the use of synergy, coherence and standardization in the process of designing production lines will reduce the costs associated with maintaining full efficiency of machines and production equipment and training technical personnel, which will result in the improvement of the enterprise’s financial indicators. During the analyses, it was observed that in addition to the reduction in costs related to the maintenance of the spare parts warehouse, the comfort of the technical team’s work also improved. It was directly influenced by standardization because technicians could be trained in the use and operation of one type of element and did not have to know the range of all products available on the market. This made it possible to narrow down the scope of competencies, which in turn resulted in the strengthening of key qualifications. Standardization was carried out on standard elements that did not require the processing of machines and devices. In the future, attempts can be made to standardize all the elements in the company, which would result in the need to make financial outlays for standardizing machine parts, but in the long run, the financial outlay would compensate for the savings that would be generated by introducing the changes described. This is a task that requires further economic analyses and functional research that would confirm the rightness of such an action. It would be necessary to group all non-standard spare parts (e.g., inverters, PLCs, motors), perform the characteristics for each element in the group, find one common replacement that we can use without losing the necessary parameters, and then adjust the machine to the possibility of using the replacement. After standardization in each group, the calculations and observations that will confirm the lack of loss of technical parameters of the device and the improvement of the company’s financial indicators should be made. After the comprehensive research in one group and the confirmation of the correctness of the actions taken, it would be possible to start working with another group. These activities will also reduce inventory levels and increase the amount of available warehouse space, which can be used in other ways, e.g., by building a training station for technical staff, where it will be possible to train new employees and improve the competencies of experienced technicians and engineers. These activities can be performed without any additional financial outlay, using their own warehouse resources. The elements on the shelves can be used to build training stations, and in the event of a failure and the need to use the selected element, disassemble it from the training station and install it in the machine. It will also allow employees to be trained and test spare parts on storage shelves. When examining the impact of the phenomena of standardization, coherence and synergy in the design of technological lines and their impact on the company’s financial indicators, the biggest challenge was the time-consuming nature of the research because, after each stage of changes, it was necessary to observe the process and its changes. The work on the research and the article lasted 1 year and 5 months (start 03.2020–end 09.2021). The results of the presented research and analyses will contribute to the development of the future planning of the spare parts warehouse economy and optimization of the maintenance costs of machines and devices in manufacturing companies. The research carried out from March 2020 to September 2021 shows that with 30% standardization of the assortment, it was possible to save approx. EUR 5263/month (Table 3), which allows the company to save approx. EUR 63 thousand per year. Assuming the trend is maintained, the company would standardize the entire warehouse, and the estimated savings would amount to approx. PLN (polish new zloty) 210.5 thousand EUR/year with 100% standardization.

## 7. Next Step of Standardization after Launching the Technological Line—eLean Implementation

The next step in the development of standardization of production processes is the implementation of the systems enabling the implementation of Lean Manufacturing. Lean Manufacturing (LM) is a management philosophy encompassing a whole set of approaches, tools and features to meet the following goals: to eliminate waste, create value in products and processes, and reduce the use of resources [50,51]. The implementation of LM also allows enterprises to increase the efficiency and flexibility of operations while reducing inventories of raw materials and finished products to simplify processes and reduce operating costs. This is especially important in the case of small and medium-sized enterprises, which have to struggle with large firms to survive in the consumer market [52,53,54,55].

A common practice in companies planning to implement Lean Manufacturing (LM) is to employ consulting companies or Lean analysts [56,57,58]. The most known consulting companies providing Lean methodology implementation services in Poland include Lean Enterprise Institute Polska, Sabat Consulting, LM Consulting Group, Leanpassion and Smart Project. The cost of the services of these companies is determined individually depending on the scope of work and the number of areas in the enterprise in which the implementation is to take place. Nevertheless, the method of providing the service consisting of the implementation of LM in the enterprise for all the consulting companies mentioned above is similar. It begins with the stage of observation of the current state, i.e., an audit, followed by consultations and advice on the possible implementation of the changes proposed by the advisor.

All the services provided by Lean advisors are based on direct observation of processes, manual data collection and manual measurements of time and other characteristics of the observed processes. This method of conducting analyses is very time-consuming, carries the risk of measurement errors and causes distortions in the course of real processes. It entails the need to involve external Lean experts for hours of observation and subsequent analysis of the results, which leads to a high cost of such a service [57,58]. The result of the prepared analyses is recommendations and solutions that the company must then implement into its business practice.

An example of such an enterprise is a company producing rock stone wool. The production of rock stone wool on which the tests were performed is a particularly complicated process consisting in melting the batch composed mainly of gabbro-basalt stones and dolomites and additives with the use of temperatures in the range of 1400–1500 °C and then spreading the lava formed after the melting of the lava rocks on the centrifuge. Due to the multi-shaft device that rotates at a given speed, the lava is thrown with centrifugal force, and a stream of cold air containing sticky resins—the binder—blows the lava and transforms it into a rock stone wool fiber. Then, the device collecting fibers in the form of a drum arranges and forms the fibers into a carpet of appropriate density and thickness, thanks to which it is possible to obtain the appropriate insulation parameters of the wool. The prepared “carpet” is then subjected to heat treatment and crystallization—polymerization. After processing, the wool is a finished product, but it is still subject to mechanical processing, such as a cross or longitudinal cutting, and it is packed.

Due to the risk of high temperature, dust or chemical substances, the process is dangerous; therefore, it must be subjected to the special supervision of control and safety devices and continuous monitoring of key parameters. Due to the use of such solutions, relatively few people work on the production process itself. The key element in the entire process is to maintain the machines and devices in constant technical efficiency because the stone wool production process is a continuous process. The optimal solution for such a process is to supervise the operation of the devices in continuous motion and periodic cleaning of the dome furnace areas—the melting furnace, polymerization furnace and all carpet collecting and laying devices. It is of particular importance to properly maintain the condition of the centrifuge, which is exposed to temperature and chemical loads. In the production company in Wykroty, the principle of continuous operation for a period of 2 weeks was adopted, followed by one day of technological downtime for the line cleaning. Once a year, a major overhaul of the production line takes place, during which all the operating elements of the line, crucial in terms of quality and efficiency, are replaced.

The plant in Wykroty belongs to the Technonicol corporation, which bases its production system philosophy on the Toyota system and implements Lean Manufacturing solutions. The 5S tool is implemented on the entire production line, but its particular application appears during service breaks every two weeks as well as during a general renovation. Due to the surface of the line, the area where individual maintenance and cleaning operations must be performed is very extensive, and the coordination of individual works is very complicated. The development of the right plan and proper allocation of resources are the main tasks of maintenance services during the operation of the line. However, despite the traditional preparations, division of duties and allocation of resources, the time for carrying out a service day or general renovation is very tight and often exceeded despite assumptions. The 5S tool definitely improves the work of maintenance services, production departments and service companies. However, the company’s management has great potential to shorten this service to the necessary minimum. At the moment, work is underway in order to define workstations during the service and general renovation days using the technological solutions of the fourth industrial revolution, especially that during the two-week production cycle, workplaces are different from those during the service. The proposed tools are tags on devices and tools intended for the service as well as defining a place for them—systematization and tracking their movement along the production line as a function of time using a local Wi-Fi network. The development of a diagram of their movement will optimize their use.

Another aspect is the readiness and availability of spare parts. Due to the dimensions and a large number of inventory indexes, the spare parts warehouse is divided into the main warehouse, a mechanical warehouse and a number of handy warehouses. The use of technological solutions with the use of presence sensors (laser, inductive, optical) will allow identification of the part in a given warehouse, confirm its availability and enable proper determination of the outflow of parts, which in turn will allow for the optimization of the purchase and delivery time as well as the minimum and optimal conditions of a given assortment. The project for the development of solutions and implementation is planned for the end of 2024, and the estimated effect will reduce downtime by at least 20%. A research model for this issue is currently being developed.

**Figure 7:**
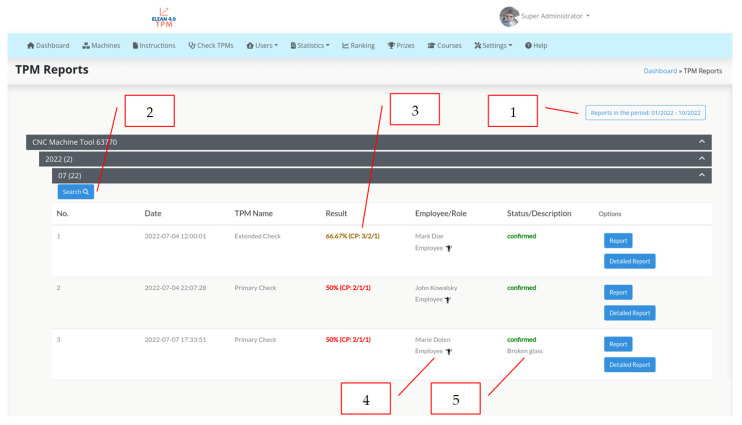
Screenshot for eLean application TPM reports.

**Figure 8:**
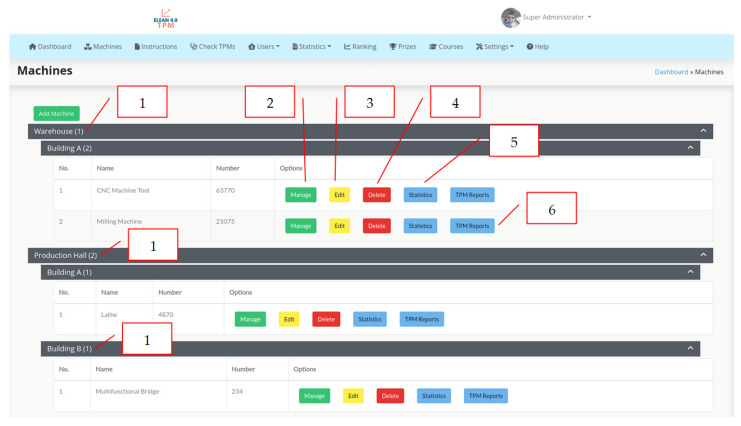
Screenshot of eLean application with an overview of machines in the company.

**Figure 9:**
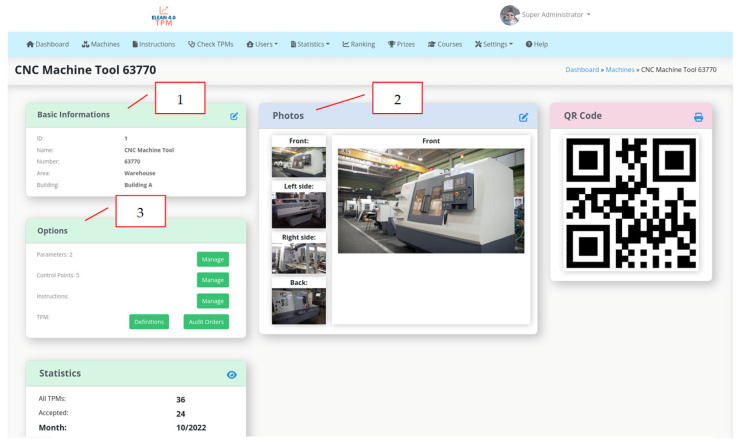
Screenshot showing the functionality of the selected eLean application—CNC Machine Tool 63770 overview.

A similar solution in the area of TPM—Autonomous Maintenance is the use of a tool that allows for an ongoing diagnosis of equipment during production. A number of elements of a production line essential from the perspective of the operation of the equipment must be diagnosed during a production process. The quality of lubrification, bending the chains or the impact of temperature on mechanical elements must be assessed in a daily, several days or a week shift period. The use of the schedule and control cards is a well-known solution in the industry that is used in other plants of the corporation. At the plant in Wykroty, the work is underway to use a technological solution from the range of industry 4.0 tools developed by the Lean Manufacturing specialists from the automotive industry and Witelon Collegium State University in Legnica and the company supporting the industry—Industrial Support. The eLean solution consists of preparing given diagnostic activities in a mobile form and using mobile devices, such as a smartphone or tablet; collecting information about the diagnosis, its duration and the result of this diagnosis; analyzing these data; and developing methods to extend the production line’s work cycle. The eLean application makes it possible to define and supervise the activities to be performed in the specific time cycles and guides a user step by step through all the defined activities. The screenshots showing the selected functionalities of the eLean application are shown below (Figure 7, Figure 8 and Figure 9).

The eLean application used to carry out the TPM checks has the functionality of searching for the reports in a given period of time for all working machines (item 1, Figure 7) and the reports for individual working machines (item 2, Figure 7). In addition to the control result expressed as a percentage (positive result above 50%), the report result is presented numerically for individual control points (CP). The first numeric value of 3 represents the number of points to be checked. Successive numerical values mean, respectively, two points compatible and one point inconsistent (item 3, Figure 7). In addition, the eLean application indicates the employee who carries out the CP (item 4, Figure 7) and specifies the statute of the control result, its confirmation and comments in the event of rejection (item 5, Figure 7).

The next screenshot of the eLean application shows a list of machines in individual areas in the enterprise (Figure 8). In this part of the application, there is a panel for managing production machines (item 2, Figure 8). It is possible to correct the information about machines by editing (item 3, Figure 8), deleting machines (item 4, Figure 8) and statistical data from the TPM control (item 5, Figure 8) and the TPM control reports for individual machines (item 6, Figure 8).

The screenshot in Figure 9 shows an example TPM control panel for a Computerized Numerical Control (CNC) machine tool 63770. The panel contains basic data about the machine (item 1, Figure 9). There are also pictures of the machine taken from the front, back, left and right (item 2, Figure 9). Additionally, the panel contains important optional data (item 3, Figure 9), such as parameters (production machine), control points and instructions; it also includes definitions (specify when, how, what and by whom it is subjected to control) and orders (generating orders is also included in the definition).

Currently, intensive work on the implementation of the eLean application in the field of TPM is underway, including at Technonicol corporation, and work on the further development of the application with new modules.

## 8. Conclusions

Taking standardization into account when designing machines and production devices has a positive impact on the economic aspects of the enterprise due to the reduction in the costs of storing spare parts and the costs of staff training (training is carried out only on selected elements used in a given area; there is no need to have knowledge of third-party solutions). All this leads to the reduction in downtime of machines and production equipment and the improvement in the efficiency of technological processes.

The sum of all elements of the process that generates the possibility of implementing standardization improves the company’s financial results and improves the comfort of work of technical staff.

Technological synergy allows for the elimination of excessive and often unnecessary warehouse resources, which, after analyzing the profits and losses, can be rationalized or completely limited, creating revenues that give the prospect of implementing the assumptions of industry 4.0 in the company. The research showed that after the implementation of the design assumptions, the costs of storing spare parts could be reduced by PLN 210,500/year

These revenues increase the chance to introduce and implement step-by-step the assumptions of the fourth industrial revolution, including the required process of automation of production and technological activities. The use of standardization in combination with technological synergy will significantly increase production efficiency while reducing production costs. In addition, unnecessary activities will be eliminated. It will result in a reduction in the cycle time in the production of products. ELean, an advanced application with a TPM module as one of its elements, can be helpful in achieving this. Its use can significantly improve the maintenance in the machine park of manufacturing companies.

The next step in the development of technology towards industry 5.0 is the development of artificial intelligence that will allow for direct cooperation between people and machines. However, the necessary aspect in the implementation of industry 5.0 will be the development of operating personnel and technical staff. Therefore, cooperation between industrial companies and universities is necessary in order to educate high-quality specialists with substantive and practical knowledge in the field of new technologies.

## Figures and Tables

**Table 1:** SWOT analysis of the possibility of introducing the assumptions of the fourth industrial revolution in production plants.

**Strengths**	**Weaknesses**
– Increasing performance. – Increasing the repeatability of the manufactured elements. – Continuous operation (without interruptions). – Minimizing vacancies (using technology in places where human work is dangerous to health). – Lowering the demand for human resources. – Reduction in costs related to working overtime. – Lowering the cost of quality control.	High implementation costs. Cannot be used for work with a high level of variability parameter. The need to train technical staff.
**Opportunities**	**Threats**
– Possibility of enterprise development. – Raising the prestige of the company. – Winning additional new markets. – Competitiveness of the enterprise in the domestic and foreign markets.	– Loss of control of devices. – No spare components on the market.

**Table 2:** Electrochemical indices characterizing corrosion processes.

Specification	Minimum Condition	Optimal Condition	Sum of the Optimal State	Cost of Purchase and Maintaining Stock
Before standardization	1 pc– inverter of the manufacturer X 1 pc– inverter of the manufacturer Y	2 pcs—inverter of the manufacturer X 2 pcs—inverter of the manufacturer Y	4 pcs of X and Y inverters in total	For 4 inverters X and Y 3200 EUR
After standardization	1 pc—X or Y manufacturer’s inverter	2 pcs—X or Y manufacturer’s inverter	2 pcs of X and Y inverters in total	For 2 inverters X and Y 1600 EUR
Difference before and after standardization	1 piece of inverter	2 pieces of inverter	2 pieces of inverter	1600 EUR

**Table 3:** Standardization of spare parts in a company from the automotive industry by manufacturer.

	Product	Weekly Costs of Spare Parts Warehouse Maintenance	Monthly Costs of Spare Parts Warehouse Maintenance	Part of the Assortment Subjected to Analysis and Standardization	Availability	Monthly/ Annual Savings
Before standardization	Spare parts (PLCs, Inverters, sensors, etc.) of two different manufacturers	8772.9EUR	35,087.7 EUR	30%	100%	none
After standardization		2631.5 EUR (10,526.3 EUR/4)	10,526.3 EUR (35,087.7 EUR ∗ 0.3)		50%	5263.1 EUR 63,157.8 EUR (10 526.3 EUR ∗ 0.5 / 5263.1 EUR ∗ 12)
Difference before and after standardization		6140.3 EUR	24,561.4 EUR		50%	29,824.5 EUR/ 357,894.7 EUR
Monthly savings	Number of months	Annual profit with standardization of 30% of the assortment	Monthly savings	Number of months	Annual profit with standardization of 10% of the assortment	
5263.1 EUR	12	63,157.8 EUR	1754.3 EUR	12	21 052.6 EUR	
Monthly savings	Number of months	Annual profit with standardization of 100% of the assortment				
17,543.8 EUR	12	210,526.3 EUR				

## Data Availability

Not applicable.
